# Results of a 20 Year Retrospective Analysis of Early-Stage Cervical Cancer: Should 3 cm Be Considered the New Ariadne’s Thread in Early Cervical Cancer Treatment?

**DOI:** 10.3390/cancers15051570

**Published:** 2023-03-02

**Authors:** Benjamin Serouart, Abel Cordoba, Carlos Martinez-Gomez, Emilie Bogart, Marie Cecile Le Deley, Éric Leblanc, Delphine Hudry, Alexandre Escande, Florence Le Tinier, Camille Pasquesoone, Sophie Taieb, Houssein El Hajj, Fabrice Narducci

**Affiliations:** 1 Department of Surgical Oncology, Oscar Lambret Center, 59000 Lille, France; 2 Department of Radiotherapy, Oscar Lambret Center, 59000 Lille, France; 3 Department of Biostatistics, Oscar Lambret Center, 59000 Lille, France; 4 Department of Pathology, Oscar Lambret Center, 59000 Lille, France; 5 Department of Imaging, Oscar Lambret Center, 59000 Lille, France

**Keywords:** cervical cancer, early stage, radical hysterectomy, minimally invasive surgery, conization, brachytherapy

## Abstract

**Simple Summary:**

Early-stage cervical cancer treatment modalities changed over the decades from open radical hysterectomy to minimally invasive surgery (MIS) and back to laparotomy after the results of a randomized controlled trial that showed a higher risk of disease recurrence in MIS. We aimed to evaluate the overall survival and recurrence-free survival and to assess disease recurrence in early-stage cervical cancer patients treated with minimally invasive surgery over a period of 20 years. Our results show that MIS can still be considered for tumors ≤ 2 cm subject to first conization followed by surgery with the Schautheim procedure and extended pelvic lymphadenectomy. We also found that tumors > 3 cm should preferably be managed with concomitant chemoradiation and brachytherapy.

**Abstract:**

(1) This study aims to evaluate the overall survival (OS) and recurrence-free survivals (RFS) and assess disease recurrence of early-stage cervical cancer (ESCC) patients treated with minimally invasive surgery (MIS). (2) This single-center retrospective analysis was performed between January 1999 and December 2018, including all patients managed with MIS for ESCC. (3) All 239 patients included in the study underwent pelvic lymphadenectomy followed by radical hysterectomy without the use of an intrauterine manipulator. Preoperative brachytherapy was performed in 125 patients with tumors measuring 2 to 4 cm. The 5-year OS and RFS rates were 92% and 86.9%, respectively. Multivariate analysis found two significant factors associated with recurrence: previous conization with HR = 0.21, *p* = 0.01, and tumor size > 3 cm with HR = 2.26, *p* = 0.031. Out of the 33 cases of disease recurrence, we witnessed 22 disease-related deaths. Recurrence rates were 7.5%, 12.9%, and 24.1% for tumors measuring ≤ 2 cm, 2 to 3 cm, and > 3 cm, respectively. Tumors ≤ 2 cm were mostly associated with local recurrences. Tumors > 2 cm were frequently associated with common iliac or presacral lymph node recurrences. (4) MIS may still be considered for tumors ≤ 2 cm subject to first conization followed by surgery with the Schautheim procedure and extended pelvic lymphadenectomy. Due to the increased rate of recurrence, a more aggressive approach might be considered for tumors > 3 cm.

## 1. Introduction

Cervical cancer is the fourth most commonly diagnosed cancer worldwide, with 604,000 new cases reported in 2020. It is also the fourth leading cause of cancer-related death in women, causing 342,000 deaths in 2020. Despite low HPV vaccination rates in France, Germany, and the United States of America (around 40%), cervical cancer incidence and mortality rates have decreased over the last three decades, attributed to effective cervical cancer screening and HPV vaccination programs [1]. 

The International Federation of Gynecology and Obstetrics (FIGO) 2018 classification categorizes early-stage cervical cancer (ESCC) as cervical tumors that are limited to the cervix (up to stage IB2) or extend to the upper two-thirds of the vagina (stage IIA1) with a maximum diameter of less than 4 cm [2].

Depending on the tumor size, fertility-sparing options could be considered in some patients; however, the recommended treatment for ESCC remains radical abdominal surgery comprising radical colpohysterectomy extended to the parametrium (RH) preceded by pelvic lymph node dissection to ensure the absence of lymph node metastasis [3,4]. The extent of this surgery, detailed by the Querleu–Morrow Classification, can be tailored to each patient according to their respective risk groups (low, intermediate, and high-risk treatment) and prognostic factors (tumor size, maximum stromal invasion, and lymphovascular space invasion—LVSI) [5].

With the development of minimally invasive surgery (MIS) and robotic assistance, MIS-RH has become the mainstream approach. This was mainly due to the reduction in morbidity and recovery time associated with MIS [6,7]. However, data from the Laparoscopic Approach to Carcinoma of the Cervix (LACC) trial concluded lower recurrence-free survival (RFS) and overall survival (OS) rates for ESCC patients treated with MIS compared to those treated with laparotomy, thus questioning the safety of the MIS approach [8]. Other studies further confirmed those results, including a meta-analysis published in 2020 [9,10,11,12]. Several hypotheses were proposed to explain these results, mainly tumor fragmentation caused by intrauterine manipulators and intrabdominal seeding caused by creating a pneumoperitoneum [13,14,15,16]. This was further confirmed by the SUCCOR study [17].

Vaginal closure using the Schauta technique before radical laparoscopic hysterectomy (Schautheim procedure) could mitigate these problems by minimizing the risk of tumor dissemination [18,19]. Several retrospective analyses and one meta-analysis concluded comparable OS and RFS rates between open radical hysterectomy and “Schautheim” laparoscopic RH [19,20,21,22,23]. 

The primary objective of this study was to evaluate the 5-year OS, and RFS of ESCC patients managed with MIS preceded for some patients by preoperative brachytherapy (POBT). The secondary objectives were assessing disease recurrence in this cohort and identifying its potential risk factors.

## 2. Materials and Methods

We conducted a single-center retrospective analysis at the Oscar Lambret tertiary cancer center in France that evaluated ESCC patients treated between 1 January 1999, and 31 December 2018. The study protocol was concordant with the French ethical standards and the 2008 Helsinki declaration. All participating patients provided written informed consent.

### 2.1. Patients Characteristics

Eligible patients were 18 years or older and managed at our center for ESCC with a tumor size < 4 cm. This included patients with 2018 FIGO stages ranging from IA1 to IB2 and IIA1. All patients underwent pelvic lymph node dissection (PLND), and those with lymph node metastasis were systematically excluded from the analysis, except for those with isolated tumor cells and micrometastases (<2 mm).

Patients presenting with tumors ≥ 4 cm or stages ≥ IIB and patients in whom initial management was performed in another center were excluded from the analysis. Other exclusion criteria included in situ disease, 2018-FIGO stage IA1 disease without LVSI, primary management with concomitant chemoradiation therapy, pathology findings in favor of non-cervical origin, management not concordant with the disease stage, and open radical surgery.

Tumor size was defined as the longest tumor diameter assessed on pathology specimens in patients who underwent conization or radical hysterectomy without POBT. In patients who underwent POBT without conization, the tumor size was evaluated clinically or based on pelvic magnetic resonance imaging (MRI). 

### 2.2. Patient Management

Before performing the RH, all patients underwent bilateral MIS PLND to confirm the absence of lymph node metastasis (external iliac LND up to the iliac bifurcation). Only a small number of patients received an additional common iliac and/or presacral lymph node dissection, as this was not recommended at the time of management of the patients included in our study.

After PLND, all patients underwent a Schauta procedure to enclose the tumor. This was followed by a nerve-sparing Querleu–Morrow type B radical hysterectomy consisting of parametrial transection at the level of the ureter after its mobilization associated with partial resection of the vesicouterine and uterosacral ligaments while preserving the vesical branches of the pelvic plexus and the hypogastric nerves, and resection of approximately 10mm of the vagina from the cervix or the tumor [24]. No intrauterine manipulator was used in any of the cases [18].

In this cohort, 125 patients with tumors between 2 and 4 cm underwent pulse dose rate POBT with a total dose of 45 Gy according to the French national recommendations and the recommendations of the European Society of Gynaecological Oncology/European Society for Radiotherapy and Oncology/European Society of Pathology Guidelines for the Management of Patients With Cervical Cancer [3]. A CT scan and/or dosimetric MRI defined the target volume beforehand.

### 2.3. Follow-Up

Overall survival was estimated from the date of initial pelvic lymphadenectomy to the date of death from any cause. Patients alive at the last count were censored at this date. Recurrence-free survival was estimated from initial pelvic lymphadenectomy to the date of first local or distant recurrence or death from any cause. Patients alive and who did not present any recurrence at the last count were censored at this date. The type of recurrence was described as local, regional, metastatic, or combined.

The prognostic value on RFS of the following factors was studied, in agreement with the literature data: patient’s age at initial management, body mass index, history of conization, presence of LVSI on initial biopsy and/or surgical specimen, histological type, differentiation grade, FIGO stage, preoperative brachytherapy as well as tumor size. The latter will be studied as a quantitative variable on the one hand and as a categorical variable, on the other hand, according to the clinically relevant thresholds, which are ≤2 cm, 2–3 cm, and >3 cm [25,26,27,28].

### 2.4. Statistical Analysis

Data analysis was conducted using Stata version 17.0 software (StataCorp. 2019. Stata Statistical Software: Release 15. StataCorp LLC, College Station, TX, USA). 

Patients’ characteristics were described using medians (minimum–maximum values), means (+/− standard deviations) for quantitative data, and frequencies (percentages) for categorical variables. Missing data were also specified. 

Since standard follow-up consists of a duration of 5 years, all estimates of efficacy criteria were truncated at this date to avoid estimation bias: deaths and recurrences that occurred more than five years after PLND were not included in this analysis, and all patients with a follow-up exceeding five years were censored at that time.

Median patient follow-up was estimated by the reverse Kaplan–Meier method. OS and RFS curves were evaluated by the Kaplan–Meier method. The OS and RFS rates with their associated 95% confidence interval (IC 95%) were given at 36 and 60 months. Univariate Cox models tested the prognostic value of the studied factors on RFS. The proportional hazards assumption of the Cox model was tested for each variable by the Schoenfeld residual test. These models’ hazard ratios (HR) were estimated with their 95% CI. The variables associated with a *p*-value < 0.20 in univariate were introduced into the first multivariate model. The final model was then constructed following a top-down stepwise procedure by retaining only the variables associated with a significant *p*-value at *p* < 0.05. 

## 3. Results

**Figure 1:**
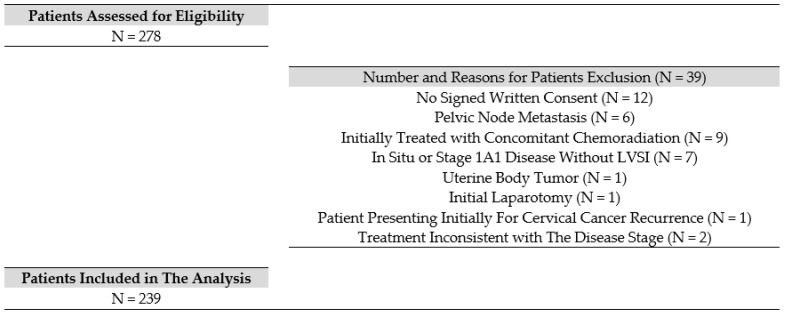
Flow chart.

Of the 278 patients assessed for eligibility, 239 met the inclusion criteria. The reasons for excluding 39 patients were as follows: the absence of a signed written consent in 12 patients; pelvic lymph node metastasis in 6 patients (i.e., advanced-stage IIIC); upfront treatment with concomitant chemoradiation in 9 patients; 7 patients presented in situ disease or stage IA1 without LVSI; 1 patient showed histology consistent with endometrial adenocarcinoma; 1 patient was managed by laparotomy; 1 patient was treated for a CC recurrence initially treated at another center; and 2 patients received treatment inconsistent with their disease stage. Figure 1 shows the flow chart of the patients included in this study, and Table 1 shows their characteristics.

In our cohort, 25% of patients underwent PLND guided by pelvic sentinel node identification, five patients underwent common iliac and presacral lymph node dissection, and an average of 20 lymph nodes were removed per patient. Nine patients presented micrometastases < 2 mm.

One hundred and twenty-five patients (52%) with a tumor size between 2 and 4 cm received POBT. The Schautheim procedure, which consists of a first protective colpotomy, was performed in 82% of the patients (i.e., first protective colpotomy), of which 74% (177 patients) were performed by standard laparoscopic approach and 24% (57 patients) by robot-assisted laparoscopic approach. A standard Wertheim procedure without protective colpotomy was performed in 16% of the patients due to the impossibility of accessing the cervix, the impossibility of performing the colpotomy, or because the surgery was performed before the description of the Schautheim procedure. All surgeries were performed without the use of an intrauterine manipulator. Conversion to laparotomy was necessary for six patients for the following reasons: ureteral dissection difficulty and suspicion of a ureteral lesion in two patients, hemorrhagic ureteral dissection in two patients, major intraperitoneal adhesions in one patient, and intolerance of the Trendelenburg position in one patient. Finally, five patients underwent fertility preservation surgeries consisting of radical vaginal trachelectomy. 

**Figure 2:**
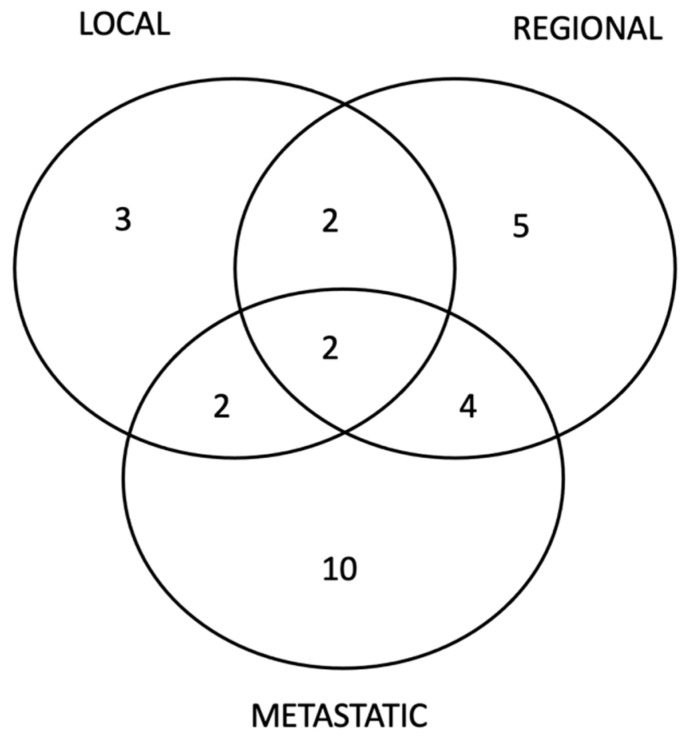
Patterns of Disease Recurrences.

A total of 17 deaths were reported in our cohort, of which 16 deaths were disease-related and 1 was non-disease-related. The OS rate was 95.1% (CI95% 91.4–97.3%) at 3 years, and 92% (CI95% 87.4–95%) at 5 years. Twenty-eight patients presented disease recurrences during the first five years: three patients presented local recurrences, five regional recurrences, ten metastatic recurrences alone, and ten combined recurrences (Figure 2). The RFS rate was 90.8% (CI95% 86.3–93.9%) at 3 years, and 86.9% (CI95% 81.6–90.7%) at 5 years. In addition to the 28 previously cited recurrences, 5 patients presented disease recurrence after the 5 follow-up years: 1 local and regional recurrence 121 months after pelvic lymphadenectomy and 4 metastatic recurrences 93, 96, 105, and 115 months after pelvic lymphadenectomy, respectively. These recurrences were not considered in the RFS estimate since it was truncated at 5 years.

**Figure 3:**
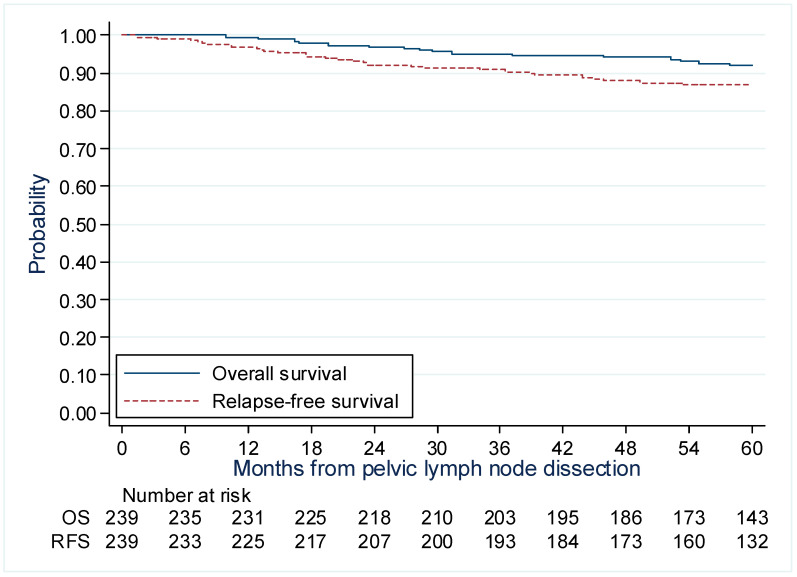
Overall and recurrence-free survival curves for patients with early-stage cervical cancer treated with surgery with or without preoperative brachytherapy.

Figure 3 shows the 5-year OS and RFS curves for patients with ESCC treated with MIS with or without POBT. The 5-year OS rate was 95.4% (86.5–98.5%) for tumors ≤ 2 cm, 92.0% (83.9–96.1%) for tumors measuring 2–3 cm, and 87.2% (75.0–93.7%) for tumors > 3 cm. The 5-year RFS rate was 89.9% (79.9–95.1%) for tumors ≤ 2 cm, 89.4% (81.3–94.2%) for tumors measuring 2–3 cm and 77.9% (64.2–86.9%) for tumors > 3 cm.

Univariate analysis showed that every 10 years increase in patients’ age was significantly associated with an increased risk of progression or death, HR = 1.37 (1.02–1.84), *p* = 0.04. Patients who underwent conization presented lower risks of progression or death compared to those who did not, HR = 0.20 (0.06–0.67), *p* = 0.009. Patients with FIGO stage II disease presented a higher risk of progression or death than FIGO stage I patients, HR = 2.87 (1.09–7.52), *p* = 0.009. Every 1cm increase in the tumor size is significantly associated with an increased risk of progression or death, HR = 1.65 (1.08–2.52) and *p* = 0.021. When tumor size was considered as a qualitative variable, we observed a monotonically increasing relationship between tumor size and HR, i.e., the value of HR increases as the category of tumor size increases: HR = 1 for lesions ≤ 2 cm, HR = 1.13 (0.43–2.96) for lesions measuring 2–3 cm, and HR = 1.60 (1.03–6.62) for lesions >3 cm, *p* = 0.06. When comparing tumors measuring > 3 cm to those measuring ≤ 3 cm, we found a significantly higher risk of progression or death, HR = 2.43 (1.16–5.09) and *p* = 0.018. The other tested factors in the univariate analysis did not show any significant association with the risk of recurrence or death. Furthermore, we did not observe a statistically significant difference according to whether or not the patient had brachytherapy and the size of the lesion ≤ 2 cm vs. >2 cm, with *p* = 0.13. 

The factors listed above were selected to establish the multivariate model because they were significant at the *p* < 0.20 threshold. Although less statistically powerful, we considered the binary stratification of the tumor size (≤3 cm versus >3 cm) for multivariate analysis because of its clinical relevance. The results of the multivariate analysis showed that the two factors significantly associated with the risk of progression or death were conization with HR = 0.21 (0.06–0.70) and *p* = 0.01, and tumor size with HR = 2.26 (1.08–4.73), *p* = 0.031 for tumors > 3 cm. The other factors selected in the initial multivariate model, such as age, stage, and POBT, did not appear significant at the *p* < 0.05 threshold and were not retained in the final multivariate model. The results of the univariate and multivariate analyses are shown in Table 2.

**Figure 4:**
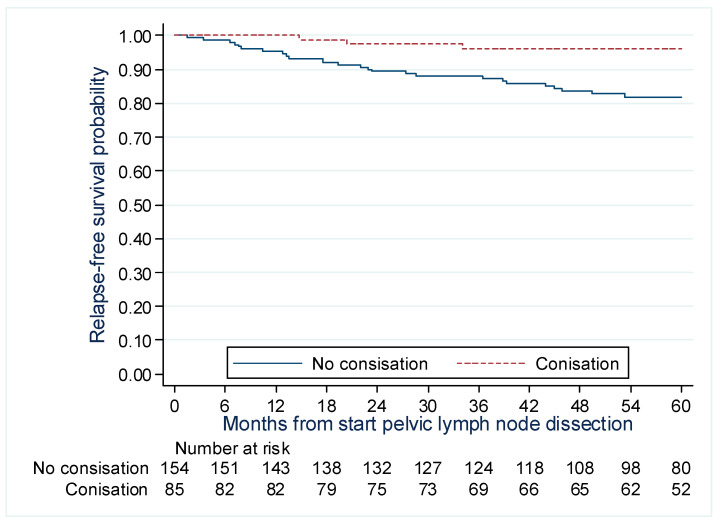
Recurrence-free survival curve by history of conization for patients with early-stage cervical cancer treated with surgery +/− preoperative brachytherapy.

**Figure 5:**
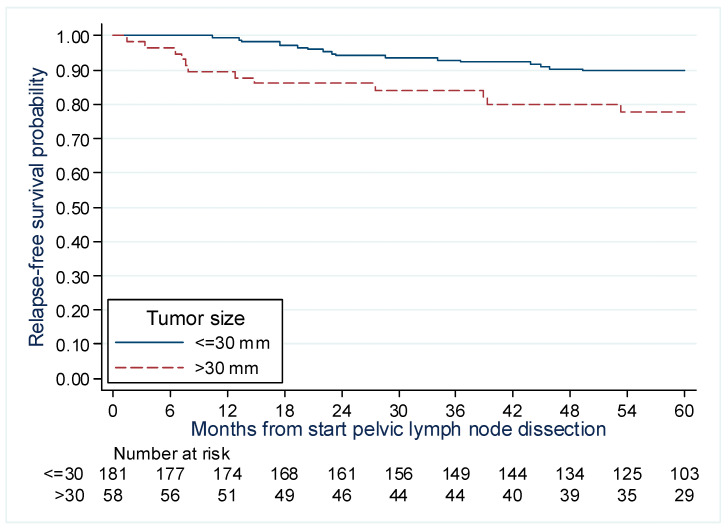
Recurrence-free survival by lesion size for patients with early cervical cancer treated with surgery +/− preoperative brachytherapy.

The significant factors identified in the multivariate model are presented in Figure 4 and Figure 5. The 5-year RFS rate was found to be higher for patients who underwent conization, at 96.1% (88.4–98.7%) compared to those who did not 82.0% (74.6–87.4%). The 5-year RFS rate for tumors measuring ≤ 3 cm was 89.7% (84.4–93.5%), whereas the rate for tumors > 3 cm was lower at 77.9% (64.2–86.9%). 

It is worth noting that there was no significant association between tumor size and conization, with a *p*-value of 0.08 from the Chi2 test.

A total of 33 recurrences were observed in the cohort, and the characteristics of each recurrence are presented in Table 3. All estimates of the 5-year efficacy criteria were censored to eliminate statistical bias in the estimates. Of the 33 patients with recurrence, 24 have passed away, 22 of whom died due to the disease.

## 4. Discussion

This retrospective study aims to evaluate the 5-year OS and RFS of a cohort of early cervical cancer patients with no lymph node involvement nor distant metastasis treated with minimally invasive surgery with or without preoperative brachytherapy. 

The European Society of Gynecologic Oncology (ESGO) recommends radical hysterectomy as the gold standard approach to treat early cervical cancer cases. However, POBT followed by surgery is also considered an acceptable strategy for selected patients to be performed only in experienced centers [3]. The NCCN guidelines recommend radical surgery or external beam radiotherapy with concomitant chemotherapy followed by brachytherapy in patients with IB1, IB2, and IIA1 disease [4].

Meticulous patient selection for cervical cancer surgery is crucial to minimize the need for adjuvant pelvic radiation therapy and its associated morbidity. This selection process should consider both clinical evaluation and imaging results. In this sense, Landoni et al. [29] published in 1997 the results of a Phase III clinical trial that compared surgery and radiotherapy in the management of early cervical cancer. They found that although both treatments resulted in similar overall or disease-free survival, the combination of surgery and radiotherapy had significant morbidity. A total of 63.5% of the patients in the surgery group required adjuvant radiotherapy due to the presence of poor prognostic factors such as positive margins, advanced stages, positive lymph nodes, massive stromal invasion, or lymph vascular invasion, indicating inadequate patient selection. The study also found a significantly higher rate of pelvic relapse among patients with tumors larger than 4 cm who received radiation alone compared to those who received surgery plus adjuvant radiation. This highlights the importance of considering both tumor size and other prognostic factors when making treatment decisions and emphasizes the need for meticulous patient selection based on clinical evaluation and imaging.

In France, radiation therapy, particularly brachytherapy, plays an important role in treating solid tumors, including cervical cancer. Several studies have evaluated preoperative brachytherapy followed by surgery in ESCC as a strategy to lower the need for adjuvant therapy and reduce urinary morbidity associated with the extended surgery; most of these studies have shown a high local disease control and a urinary morbidity rate approaching 5% [7,30,31,32,33,34,35,36,37]. Currently, preoperative brachytherapy is only recommended for patients presenting 2018 FIGO stage IB2 disease [2,3].

The size of the tumor has always been considered a crucial prognostic factor for local control of cervical cancer. Prior to the new 2018 FIGO classification, the IB1 tumors consisted of a very heterogenous subcategory of tumors with a wide range of presentations. In our previous publication [30], we presented the outcome of 80 ESCC patients with tumors measuring 2 to 4 cm treated with POBT followed by surgery. Our results showed that patients with tumors measuring 3 cm or more had worse 5-year disease-free survival than tumors measuring less than 3 cm. Escande et al. [31] came to the same conclusion in their retrospective cohort evaluating 160 cases of ESCC treated with POBT followed by surgery. In this study, the two factors associated with a lower risk of recurrence and enhanced overall survival were previous conization, regardless of the tumor size, and a tumor size of less than 3 cm.

### 4.1. Conization

Our results show a protective effect of conization before surgery, with an enhanced 5-year RFS rate of 96.1% (88.4–98.7%) compared to 82.0% (74.6–87.4%) for patients without previous conization. In the SUCCOR study, Chacon et al. [38] concluded a 75% reduction in the risk of death HR = 0.25, 95% CI (0.07 to 0.90), *p* = 0.033 and a 65% reduction in the risk of relapse HR = 0.35, 95% CI (0.16 to 0.75), *p* = 0.007 in ESCC who underwent cervical conization prior to radical hysterectomy. These results could be interpreted as a result of better measurement of the tumor size before planning radical surgery and the decreased risk of tumoral spillage.

Casarin et al. [26] evaluated the outcomes of laparoscopic radical hysterectomy in ESCC patients and found that preoperative conization was significantly associated with lower risks of recurrence 1/93 (1.1%) compared with 15/93 (16.1%) in patients who only underwent cervical biopsy (*p* < 0.001). The sub-analysis of the stage IB1 subgroup concluded the same results: the recurrence rate was 1.8% compared with 17.2% in patients who underwent preoperative conization and cervical biopsy, respectively; *p* = 0.004. In line with these results, Bizzarri et al. [39] showed in their retrospective analysis that a better 5-year disease-free survival (DFS) rate of 89.8% was found in patients who underwent conization before surgery compared with 80.0% in patients who did not; *p* = 0.010. The tumor size > 20 mm (HR (95% CI) 0.438 (0.232–0.825), *p* = 0.011) and the absence of conization (HR (95% CI) 2.151 (1.143–4.050), *p* = 0.018) were identified as factors independently related to a higher risk of recurrence. 

### 4.2. Mini-Invasive Surgery versus Open Surgery and Tumor Size

Our results show different patterns of recurrence depending on the tumor size: for tumors < 2 cm, disease recurrences are mostly local with a very low risk of regional and metastatic recurrence; for tumors between 2 and 3 cm, recurrences were mostly regional, and for tumors measuring more than 3 cm recurrences were mostly metastatic. The RFS rate was 90.8% (CI 95% 86.3–93.9%) at 3 years and 86.9% (CI 95% 81.6–90.7%) at 5 years. 

Landoni et al. [40] prospectively compared the outcomes of performing Piver I—simple hysterectomy or Piver III—radical hysterectomy in ESCC. They found no differences in the survival and recurrence patterns in patients with FIGO stages IB-IIA < 3 cm. Those results were not applicable for larger tumors that required adjuvant treatment in 62.4% of patients: 69% of the patients treated with Piver I hysterectomy required radiotherapy compared with 55% of the Piver III group. Corrado et al. [41] retrospectively compared the different approaches to treating ESCC. The rates of required adjuvant radiotherapy were 24 (23.7%), 35 (23%), and 24 (27.2%) for the laparotomy, standard laparoscopy, and robotic-assisted MIS groups, respectively. The estimated 5-year RFS rates were 91.3%, 87.2%, and 89.5% for the laparotomy, standard laparoscopy, and robotic-assisted MIS groups, respectively. A tumor size > 2 cm seemed to be associated with an increased risk of recurrence. Melamed et al. [9] evaluated in their retrospective analysis 2461 ESCC patients and concluded higher rates of mortality in MIS (HR 1.65 CI 95% 1.22–2.22). However, these findings were statistically significant in tumors measuring < 2 cm, HR 1.46 (CI 95% 0.70–3.02).

The LACC trial is one of the most impactful studies in the ESCC [8]. Ramirez et al. randomized 631 patients to either open surgery or MIS; the adjuvant treatment rate is similar in both groups (open-surgery group: 28.8% and minimally invasive group: 27.6%); 3-year DFS was 91.2% in the MIS group and 97.1% in the open-surgery group (HR 4.74, 95% CI 1.63 to 8.58). In this study, patients who underwent laparoscopic colpotomy had an increased risk of locoregional recurrence (16% vs. 5%), particularly in the form of peritoneal carcinomatosis (62% of cases). 

This trial underwent several criticisms, including using a vaginal manipulator in MIS that can favor vaginal contamination and increase the number of local and regional recurrences; in their initial report, they did not evaluate survival rates according to the tumor size. The latest published data by Ramirez et al. and Melamed et al. show no significant difference in terms of disease recurrence for tumors less than 2 cm; however, these studies did detail the use of an intrauterine manipulator, protective colpotomy, or previous conization.

We think that the absence of the use of an intrauterine manipulator associated with the systematic protective colpotomy and the frequently performed pre-operative conization in our cohort might explain the accordance of our survival data with those of the LACC trial and the data published by the Canadian cervical cancer collaborative for tumors measuring < 2 cm.

The Canadian cervical cancer collaborative [42] published their retrospective cohort of patients treated for FIGO stages IA1 and IA2 cervical cancers treated by surgery between 2007 and 2019. Patients underwent open surgery, minimally invasive surgery, or combined vaginal-laparoscopic hysterectomy. Their results show no significant differences in the 5-year relapse-free survival (93.7%, 96.7%, and 90.0%, respectively, *p* = 0.34).

SUCCOR study [17] evaluated 693 patients retrospectively who underwent a radical hysterectomy by open or minimally invasive surgery for stage IB1 cervical cancer, and they did not find differences in terms of relapse rates in tumors of less than 2 cm without the use of a vaginal manipulator; in tumors larger than 2 cm they observed a deleterious impact of mini-invasive approach (HR 2.31; 95% CI 1.37–3.90); adjuvant treatment was delivered in 236 patients (58.71%) with an open surgery arm versus 137 patients (47.08) with a mini-invasive arm, *p*: 0.002. 

The previously cited data from the literature and the results of our retrospective analysis confirm that the debate on the most appropriate surgical approach for ESCC is still open. In this sense, the recommendations published by the French Collaborative Group of Gynecological Surgery (GINECO) for managing early-stage cervical cancer with minimally invasive surgery [13] state that in case of using the laparoscopic/robot approach, it is preferred not to use an intrauterine manipulator. They also recommended performing the colpotomy via vaginal approach to better define the length of vaginal resection and decrease the risk of tumoral dissemination.

### 4.3. Strengths and Limitations

Our study has limitations; notably, its retrospective and mainly monocentric nature, which makes us cautious while interpreting our results. Additionally, there is a lack of data on stromal infiltration and mild and moderate toxicity related to brachytherapy, and only severe toxicity data is available. However, it also has the advantage of presenting a large retrospective cohort over 20 years. The concordance of our data with those of the literature, particularly those of prospective multicenter studies such as the LACC or EMBRACE-I trial, is another factor that makes us reconsider how to tailor management for patients with early-stage CC better. Our practices concerning the patients with micrometastases were in line with data from the SENTICOL 1 and 2 study, but this is no longer the current state of the art, and we should await the results of the SENTICOL 3 study before drawing conclusions.

## 5. Conclusions

The results of this study suggest that the two factors independently associated with recurrence in early stages cervical cancers were the absence of previous conization and tumor size exceeding 3 cm. MIS can be considered a treatment option for tumors ≤ 2 cm if these patients underwent previous conization and a Shautheim procedure. However, given the high risk of recurrence (24.1%) after POBT and surgery for tumors ≥ 3 cm, a potential paradigm shift should be considered by adopting a more aggressive approach or maybe lowering the threshold for locally advanced cancers to 3 cm instead of 4 cm. For tumors between 2 and 3 cm, the recommended approach remains surgery by laparotomy. Surgery and BT can only be performed within a clinical trial.

## Figures and Tables

**Table 1:** Characteristics of the patients and the disease (N = 239).

Age		
Median-(Range)	46	(25; 80)
Mean-SD	47.6	11.6
BMI in kg/m²		
Median-(Range)	23.9	(15.2; 46.4)
Mean-SD	25.6	5.8
History of conization		
No	154	64.4%
Yes	85	35.6%
Presence of LVSI		
No	190	79.5%
Yes	49	20.5%
Histological type		
Epidermoid	172	72.0%
Adenocarcinoma	60	25.1%
Epidermoid and Adenocarcinoma	5	2.1%
Glassy cell	2	0.8%
Grade of Differentiation		
Low	78	32.6%
Medium	63	26.4%
Well Differentiated	98	41%
Largest diameter in cm		
≤2	80	33.5%
2 to 3	101	42.3%
>3	58	24.3%
FIGO Stage		
IA2	10	4.2%
IB1	70	29.3%
IB2	141	59.0%
IIA1	18	7.5%

**Table 2:** Association between RFS and characteristics of patients with early-stage cervical cancer treated with surgery +/− preoperative brachytherapy (N = 239).

Association between Prognostic Factors and Recurrence Free Survival	Number of Events/N	Univariate Analysis			Multivariate Analysis		
		HR	95% CI	*p*-Value	HR	95% CI	*p*-Value
Age at treatment				0.04			NS ^(2)^
HR/10 years	-	1.37	1.02–1.84		NS		
Body Mass Index				0.28			-
HR/1 kg/m²	-	1.03	0.97–1.09		-		
History of Conization				0.009			0.01
No	26/154	1			1		
Yes	3/85	0.20	0.06–0.67		0.21	0.06–0.70	
Lymphovascular space invasion				0.99			-
No	23/190	1			-		
Yes	6/49	1.00	0.41–2.46		-		
Histological type				0.49			-
Squamous cell	23/172	1			-		
Adenocarcinoma	5/60	0.56	0.21–1.47		-		
Other ^(1)^	1/7	1.03	0.14–7.63		-		
Grade of Differentiation				0.29			-
Low	10/78	1			-		
Middle	10/63	1.33	0.55–3.18		-		
High	8/97	0.63	0.25–1.59		-		
Stage				0.03			NS ^(2)^
I	24/221	1			NS		
II	5/18	2.87	1.09–7.52		NS		
Size of the Lesion				0.021			- ^(3)^
HR/10 mm	-	1.65	1.08–2.52		-		
Size of the Lesion				0.06	-		
≤20 mm	7/80	1			-		
(20–30] mm	10/101	1.13	0.43–2.96		-		
>30 mm	12/58	1.60	1.03–6.62		-		
Size of the Lesion				0.018			0.031
≤30 mm	17/181	1			1		
>30 mm	12/58	2.43	1.16–5.09		2.26	1.08–4.73	
Brachytherapy				0.066			NS ^(2)^
No	9/114	1			NS		
Yes	20/125	2.09	0.95–4.59		NS		
Size of the lesion and Brachytherapy				0.16 ^(4)^			
size ≤ 20 mm without brachytherapy	7/75	-			-		
size ≤ 20 mm with brachytherapy	0/5	-			-		
size >20 mm without brachytherapy	2/39	-			-		
size > 20 mm with brachytherapy	20/120	-			-		

NE = Not estimated. ^(1)^ The histological types “squamous cell and adenocarcinoma” (N = 5) and “glassy cell” (N = 2) were grouped together under the term “other” because they are not well represented. ^(2)^ NS = not significant. Adjusted on the final multivariate model, the following factors were not significant: Age: HR = 1.25 (0.92–1.70), *p* = 0.16. Stage: HR (stage I) = 1, HR (stage II) = 1.95 (0.73–5.24), *p* = 0.19. Brachytherapy: HR (no brachytherapy) = 1, HR (brachytherapy) = 1.37 (0.58–3.21), *p* = 0.47. ^(3)^ If lesion size had been considered as a quantitative variable for multivariate analysis, the result in a multivariate model including the history of conization and tumor size would have been HR/10 mm = 1.29 (1.01–2.52), *p* = 0.046. ^(4)^ The *p*-value indicated corresponds to the log-rank test. The HR and *p*-value from the Cox model cannot be estimated because no events were observed among the 5 patients with a lesion ≤ 20 mm with brachytherapy.

**Table 3:** Characteristics of disease recurrence (N = 33).

Age	Size (mm)	Figo Stage	Histology	Grade	Preoperatory Conization	Invasion of the Paracervix or Vagina	Lympho-Vascular Space Invasion	Status of Pelvic Nodes	Preoperatory Brachytherapy	Adjuvant Treatment	Time to Recurrence (Months)	Site of Recurrence	Treatment of Disease Recurrence	Death
50	20	IB2	AC	G1	No	No	Non	Negative	No	No	35.9	Aortic node	CT + RT	No
55	20	IB2	ASC	G1	No	No	Yes	Negative	No	Yes	22.6	Centro-pelvis	CT	Yes
74	19	IIA1	SCC	G2	No	Yes	Yes	Negative	No	No	50.2	Vagina	RTCT	Yes
62	20	IIA1	SCC	G1	No	Yes	Non	Negative	No	Yes	23.7	Vagina and lung	BT + CT	Yes
59	14	IIA1	SCC	G2	No	Yes	Non	Negative	No	No	17.8	Vagina	S + RT	No
41	12	IIA1	SCC	G1	No	Yes	Non	Negative	No	No	23.3	Vagina	S + RT	No
57	28	IB2	SCC	G1	No	No	No	Negative	Yes	No	18	Lung	S + CT + RT	Yes
52	24	IB2	SCC	G3	No	No	No	Negative	Yes	No	15	Para-rectal and lung	S + CT	Yes
64	30	IB2	SCC	G2	No	No	No	Negative	Yes	No	11.5	Iliac node	RTCT	Yes
67	30	IB2	SCC	G1	No	No	No	Negative	Yes	No	105	Lung	S	No
44	25	IB2	SCC	G1	No	No	Yes	Negative	No	Yes	115.9	Node, lung, and bone	CT + RT	Yes
63	30	IB2	SCC	G2	No	No	No	Negative	Yes	No	34.6	Iliac node and lung	S + CT	Yes
38	21	IB2	AC	G3	No	No	Yes	Negative	No	Yes	96.6	Peritoneal carcinosis	S + CT + RT	No
44	23	IB2	SCC	G3	No	No	Yes	Negative	Yes	No	10.5	Sacrum node	RTCT	Yes
38	25	IB2	SCC	G3	Yes	No	Yes	Negative	Yes	No	33	Iliac node	RTCT	No
55	25	IB2	AC	G2	Yes	Yes	No	Negative	Yes	No	42.1	Node, lung, and bone	CT	Yes
46	24	IB2	AC	G3	No	No	Yes	Negative	Yes	No	7.7	Vagina and node, lung	S + RTCT	No
63	30	IB2	SCC	G3	No	No	No	Negative	Yes	No	26.8	Lung	CT	Yes
53	28	IB2	SCC	G3	No	No	No	Negative	Yes	No	10.3	Lung, bone, and cerebral	CT	Yes
64	39	IB2	AC	G1	No	No	No	Negative	Yes	No	121.2	Iliac node and rectum	S	No
39	38	IB2	SCC	G2	No	No	No	Negative	Yes	No	25.8	Iliac node and sigmoid	S + RTCT	Yes
53	35	IB2	SCC	G2	No	No	No	Negative	Yes	No	4.7	Iliac node	S + RTCT	Yes
54	32	IB2	SCC	G1	No	No	No	Negative	Yes	No	93.6	Lung and bone	CT	Yes
52	33	IB2	SCC	G3	No	No	No	Negative	Yes	No	4.2	Peritoneal carcinosis	CT	Yes
48	39	IB2	SCC	G2	No	No	No	Negative	Yes	No	10.7	Iliac node and bone and lung	CT	Yes
48	39	IB2	AC	G1	No	No	No	Negative	Yes	No	36.4	Lung	CT	Yes
40	39	IIA1	SCC	G2	No	Yes	Yes	Negative	No	No	2.5	Left side pelvis	S + CT	No
62	37	IB2	SCC	G2	No	No	No	Negative	Yes	No	6.2	Iliac node and lung	CT + RT	Yes
38	34	IB2	SCC	G1	No	No	No	Negative	No	No	3.4	Iliac node and *p* carcinosis	CT + RT	Yes
54	35	IB2	SCC	G3	No	No	No	Negative	Yes	No	39.4	Node, bone, and lung	CT	Yes
38	34	IB2	SCC	G3	No	No	No	Negative	Yes	No	4.1	Peritoneal, bone, and lung	CT	Yes
70	36	IB2	SCC	G3	No	No	No	Negative	Yes	No	52	Lung, hepatic, and bone	CT	Yes
49	36	IB2	SCC	G3	Yes	No	No	Negative	Yes	No	12.4	Iliac node and lung	CT	Yes

AC = adenocarcinoma; SCC = squamous cell carcinoma; ASC = adenosquamous carcinoma; S: surgery; RT: radiotherapy; CT: chemotherapy; RTCT: concurrent chemoradiation; BT: brachytherapy.

## Data Availability

The data presented in this study are available on request from the corresponding author.
